# Parkinson’s disease beyond motor symptoms: a review of the validity and limitations of current classification of non-motor symptoms

**DOI:** 10.3389/fpsyt.2026.1831447

**Published:** 2026-06-08

**Authors:** Asad Makhani, Tiago A. Mestre, Anne-Mary Abe

**Affiliations:** 1 Department of Medicine, University of Ottawa, Ottawa, ON, Canada; 2 Parkinson’s Disease and Movement Disorders Clinic, Division of Neurology, Department of Medicine, University of Ottawa, Ottawa, ON, Canada; 3 Department of Psychiatry, The Ottawa Hospital Research Institute, Ottawa, ON, Canada

**Keywords:** emerging classifications, neuropsychiatric symptoms, NMS classification, non-motor symptoms, Parkinson’s disease

## Abstract

Non-motor symptoms (NMS) in Parkinson’s disease (PD) are highly prevalent and significantly impact quality of life. However, they are under-recognized and undertreated. Current classification systems of NMS inadequately reflect the phenomenological complexity and interrelationship of these symptoms. In this narrative review, we synthesized recent evidence about NMS in PD to critically appraise the current symptom-based classification. We also explore emerging classification models that may better capture the multifaceted nature of these symptoms.

## 1 Introduction

Non-motor symptoms (NMS) of PD are very common and contribute to poor quality of life (QoL) for the patient and their caregivers (1), yet NMS remain underrecognized in clinical practice (2). In a large multi-center study, Zis et al. (3) found NMS to be common amongst drug-naïve PD patients with approximately 45% of them experiencing severe to very serve burden of NMS (3). There has been an increased interest in recent times with new studies characterizing their prevalence, neurobiology, and management of NMS, underscoring their importance in the lived experience (4–8).

NMS symptoms include anxiety and affective symptoms, cognitive impairment, autonomic dysfunction, sensory abnormalities, circadian rhythm disruptions, hyposmia, and sleep disorders (9, 10). REM sleep behavior disorder (RBD), constipation and hyposmia are examples of NMS that can precede the emergence of parkinsonism as part of pre-motor prodrome. In PD (11, 12) NMS may follow distinct temporal trajectories across the natural history of PD course, with some emerging early and others becoming more prominent at a later stage (12, 13). The number of NMS seems to have a greater influence on QoL than motor symptoms in patients with PD across all stages of the disease (3, 14).

Symptom classifications in PD and diagnostic categories are helpful to standardize language for effective communication between clinicians, with patients, and clinical researchers. Most clinical and research frameworks classify NMS into discrete domains, such as mood, cognition, sleep, autonomic function, and sensory abnormalities (8, 15, 16). While this structure is practical, it may not adequately reflect the complexity and disease heterogeneity of NMS presentation (4). Many of the NMS, such as fatigue and apathy can overlap clinically and, in at least some domains, are linked to common neural circuits and neurotransmitter systems (8, 17).

This narrative review provides a description of NMS and examines the current classification system and their adequacy to capture the phenomenology and physiopathology of NMS in PD.

## 2 Methods

We conducted a targeted search of the English-language peer-reviewed literature using PubMed and MEDLINE, with the last search conducted on May 1, 2026, and limited to the last 5 years (up to 2021). Search terms included “Parkinson’s disease,” (“non-motor symptoms,” OR “NMS”), each individual symptom domain, together with (“management” OR “pharmacological” OR non-pharmacological”),” “machine learning,” “subtype,” (“Parkinson’s disease AND trajectory”), and (“Parkinson’s disease AND classification”). We prioritized recent reviews (last five years) and landmark studies. Given the narrative nature of this review, no other inclusion or exclusion criteria or quality assessment was applied. Additional relevant studies were identified through the reference lists of the included manuscripts and included in the current review by consensus between authors.

## 3 Current frameworks for the classification of non-motor symptoms in PD

### 3.1 Symptom-based classification

Parkinson’s disease (PD) was first described in 1817 by London physician James Parkinson in his essay *“The Shaking Palsy.”* (18) In his essay, he described progressive motor symptoms but also documented NMS, including sleep disturbances, gastrointestinal symptoms, bladder dysfunction, in addition to delirium and manifestations suggestive of psychosis (18). Decades later, Jean-Martin Charcot further characterized the condition, identifying the core feature of bradykinesia and proposed the eponym “Parkinson’s disease” (19). As the understanding of PD evolved, the recognition of a diverse symptomatology led to the categorization of NMS into distinct domains, such as autonomic dysfunction, cognitive decline, neuropsychiatric disturbances, sleep disorders, and sensory deficits (4). This framework is reflected in commonly used assessment tools, including the Movement Disorder Society - Unified Parkinson’s Disease Rating Scale (MDS-UPDRS) (20), Non-Motor Symptoms Scale (NMSS) (21), and Nonmotor Symptoms Questionnaire (NMS-Quest) (22).

Herein, we provide a synthesis of NMS in PD, with emphasis on its prevalence and known pathophysiology to reflect the adequacy of symptom-based classification to describe NMS in PD.

#### 3.1.1 Hyposmia

Hyposmia is one of the earliest symptoms of PD, presenting at least four years (up to decades) prior to the onset of motor symptoms and remains stable during the course of the disease (23). It is considered one of the cardinal NMS of PD, being present in up to 90% of patients (23–25). Braak et al. proposed six stages of PD progression, postulating the start of PD pathology in the olfactory bulb and/or the dorsal motor nucleus of the glossopharyngeal and vagal nerves based on post-mortem data (26, 27). The interaction of an environmental vector, including air pollutants (28, 29) or neuropathic pathogens, via the olfactory system (30, 31) is thought to contribute to the risk of developing PD. Hyposmia is one of the strongest clinical predictors of underlying α-synuclein pathology (32, 33).

#### 3.1.2 Pain

Pain is the most common sensory symptom in PD, reported in up to two-thirds of patients (34). In PD, pain is commonly located in the lower back and lower limbs (35). In his paper Pain in Parkinson’s Disease, Ford (36) provides a clinical classification of painful/unpleasant sensations in PD into musculoskeletal, radicular/neuropathic, and central or primary pain categories (36). The basal ganglia, along with the locus coeruleus, raphe nucleus, thalamus and the spinothalamic tracts, are involved in pain modulation and perception (37). Dopaminergic dysfunction in the nigrostriatal pathway has been implicated in pain hypersensitivity (37). Although dopamine influences pain perception, dopaminergic treatments are not always effective analgesics in PD, suggesting roles of serotonin, noradrenaline, and other neurotransmitters in central pain modulation (38).

#### 3.1.3 Depression

Depression, often comorbid with anxiety, affects approximately 40% of PD patients, with higher rates observed in females, those with a younger age of onset of PD, longer duration of the disease, and individuals with lower education levels (39). Mood changes can precede motor symptoms by up to five years (40) and are strongly associated with reduced QoL across all disease stages (41, 42). The pathophysiology of depression in PD involves serotonin, dopamine, and noradrenaline neurons outside of the nigrostriatal pathways (41, 43, 44). Lower levels of dopamine in the striatum and limbic regions, alongside increased levels of noradrenaline and denervation in the thalamus, limbic, and locus coeruleus have been noted in PD patients with depression (43, 45). Changes to serotonergic, dopaminergic, and cholinergic pathways also occur during significant life events and contribute to the development of mood disorders in PD (45).

#### 3.1.4 Anxiety

The risk of anxiety in PD is twice of the general population with approximately 30% of PD patients reporting anxiety (46, 47). Female sex, younger age, and more severe disease are considered to be risk factors for anxiety (48). Broen et al. (49) argue that the *Diagnostic and Statistical Manual* (DSM) criteria for anxiety disorders may not adequately describe the symptoms of anxiety reported in patients with PD (49). Starkstein et al. (50) classified anxiety as either “episodic” or “persistent” in PD (50). Examples of episodic anxiety include symptoms of panic disorder, agoraphobia, and social phobia (50). Persistent anxiety consists of symptoms of generalized anxiety and has a strong correlation with symptoms of comorbid depression (49).

In their study, Broen et al. (49) also reported the association of disabling and impairing disease motor-related features and episodic anxiety (49). For example, patients who experience freezing of gait, are more likely to report panic attacks (49).The study also notes that other factors, such as fear of falling, may also contribute to increased anxiety levels (49). Worsening of symptoms that accompany disease progression has been associated with increased avoidance behavior, which may contribute to social withdrawal (49). There is inconclusive evidence whether daily doses of dopaminergic medications, such as levodopa, are a risk factor for anxiety in PD (49).

The serotonergic system, particularly through the pre- and post-synaptic 5-HT1A receptors, significantly influences anxiety and its neurobehavioral manifestations in PD. Activation of these receptors reduces serotonin synthesis, turnover, and release, modulating neurotransmitters such as GABA, glutamate, and dopamine, thereby contributing to the neuropsychiatric complications associated with serotonin depletion in various brain regions, including the caudate, hypothalamus and frontal cortex (51). An International Parkinson and Movement Disorder Society (MDS) review of treatment options for non-motor symptoms (2025) notes that there is a paucity of high-quality research that investigates the management of anxiety disorders in PD as a primary outcome (52).

#### 3.1.5 Apathy

Apathy is defined as a lack of motivation characterized by “reduction of goal-directed behavior,” (53) and often involves a lack of interest, emotion, or motivation (54). In the MDS review (2015), the prevalence of apathy was noted to be approximately 40% in PD (55), of which 43% of cases do not have concurrent depression, suggesting apathy can less commonly occur independently (55). Nevertheless, apathy is associated with older age, worsening of cognitive function, depression, increased motor symptoms, and more severe disability (55). Béreau et al. (56) identified two types of apathy: 1) motivational, driven by impairment in the serotonergic and dopaminergic pathways, and 2) cognitive, associated with norepinephrine and acetylcholine dysfunction in the dorsolateral prefrontal cortex and caudate nucleus (56).

#### 3.1.6 Fatigue

Fatigue is present throughout the natural history of PD since the pre-motor phase and becomes a more common symptom during the later stages of PD (57). Overall, it is present in approximately 50% of patients with PD (58). Fatigue is associated with older age, longer disease duration, higher levodopa dose, higher severity of parkinsonism (UPDRS-III motor scores), more severe cognitive impairment as measured by (Mini-Mental State Examination/MMSE) and higher levels of anxiety and depressive symptoms (59).

Fatigue in PD may be driven by neurobiological processes that are partly independent of motor symptom severity (60, 61) and distinct from excessive daytime somnolence (61). Sicillano et al. (59) hypothesize that fatigue in PD may result from disruption of non-dopaminergic pathways, as suggested by a lack of association between fatigue and motor symptoms. They also note alterations in serotonergic signaling, as evidenced by a decrease in serotonin transporter in the basal ganglia and limbic structures in PD, which may contribute to dysfunction of frontal-basal ganglia circuitry and impaired integration of limbic input and motor functions (59). Other hypotheses include involvement of proinflammatory cytokines, prefrontal pathology, and the autonomic nervous system (62). A MDS review (2019) concluded that the management of fatigue is complex (15), reflecting the complexity of the networks involved.

#### 3.1.7 Cognition

The point prevalence of dementia in PD is approximately 30% (63). The Sydney cohort, have shown that 83% of PD patients develop dementia 20 years after diagnosis (64). The presence of neocortical Lewy bodies is highly associated with dementia in PD (65). The distribution of α-synuclein positive cortical Lewy bodies, particularly in the limbic and paralimbic regions such as the parahippocampal region, may have more clinical relevance than the overall Lewy body burden (66–68). The degeneration of cholinergic pathways and dopaminergic tracts forms the primary pathophysiological basis for dementia in PD (69). Cortical thinning in the occipito-parietal, fusiform, premotor, precuneus, temporal, and prefrontal cortices in the right hemisphere have been shown to correlate with stages of PD (70). As patients progress from early PD to moderate PD, and PDD, the level of cortical involvement increases, which is consistent with Braak’s hypothesis of PD pathological progression (27). In patients with Mild Cognitive Impairment (MCI), working memory, executive function, language, memory, and visuospatial deficits may be impacted (71). Though these impairments are not sufficient to significantly impair functioning, they mark the beginning of progressive cognitive decline (72). These impairments arise from disruptions in dopamine-dependent fronto-striatal pathways, with nigrostriatal and mesocortical pathways playing prominent roles (73, 74). Alzheimer’s disease is comorbid and clinically relevant in patients with PD. Autopsy studies confirm the presence of tau and amyloid-ß to be severe enough to affect cognition and further add to the cognitive decline in patients with PD (75). Accumulation of ß-amyloid has been associated with more rapid cognitive decline in this population (76, 77). Furthermore, cerebrovascular and small vessel brain disease have been identified in PD and may contribute to cognitive impairment (78).

#### 3.1.8 Psychosis

PD Psychosis (PDP) presents in a clinical spectrum ranging from benign illusions (misinterpretation of an object), to well-defined visual hallucinations (abnormal perception without physical stimulus), and delusions (fixed, false beliefs) (79, 80). Common themes of delusions include paranoia, including spousal infidelity, grandiosity, somatic, persecutory, and religious delusions (81). Patients who develop PD at a younger age are associated with a greater risk of developing delusions (82). Risk factors contributing to psychosis include disease duration, disease severity, cognitive impairment, and disturbances in the sleep cycle (83, 84). Hallucinations are particularly significant as they represent one of the highest risk factors for long-term care placement (85). Acetylcholine denervation has been implicated in the development of hallucinations, potentially through the promotion of REM imagery during wakefulness (86–88). While psychosis in PD can be precipitated or worsened with dopaminergic treatment, it may also occur independently of these medications (89, 90).

PDP is associated with disease-related disruptions in neural systems involved in visual perception, sensory integration, reality monitoring, and attention (91). Cortical cholinergic denervation and increased 5-HT2A serotonergic receptor binding in the ventral visual pathway, medial orbitofrontal cortex, and insula play a role in the development of visual hallucinations (92). A key advancement in PDP treatment is the development of drugs targeting cortical postsynaptic 5-HT2A receptors, such as pimavanserin (92, 93). Clozapine, an antipsychotic with strong 5-HT2 receptor affinity has also been shown to be effective in treating PDP (93).

#### 3.1.9 Sleep

Sleep disorders affect approximately 90% of patients with PD (94). Changes in hypocretin (orexin), a hormone regulating sleep, has been implicated in PD sleep disturbances, mirroring findings in narcolepsy patients who experience excessive daytime sleepiness (95). Schutz et al. (96) highlight various other factors that may also contribute to sleep disturbance in PD, including motor symptoms of akinesia and tremor, and NMS such as autonomic or neuropsychiatric symptoms, side effects of dopaminergic or concurrent medication, primary sleep disorders, dysfunction of circadian rhythm, and neurodegeneration of sleep regulatory pathways such as the hypothalamus and brainstem. Sleep disturbances in PD include insomnia, excessive sleepiness, periodic limb movements, circadian rhythm abnormalities and RBD (97). RBD is one of the strongest clinical prodromal marker of synucleinopathy and is associated with the development of PDD or DLB (98). Neuropathology degeneration of the orexinergic neurons in the hypothalamus, in addition to dopaminergic neuron loss contribute to the sleep dysfunction in PD (99). However, orexinergic neuron degeneration occurs after the dopaminergic neurons are affected (99). Orexin has a role in signaling arousal to counter potential or actual hazards, and its deficiency leads to a decoupling of arousal from internal and external cues, resulting in sleep-wake instability (100). Orexin deficiency has also been linked to cataplexy and REM behavior disturbances (101). Sleep disturbances in PD are therefore heterogeneous in pathology.

The present review of NMS in PD highlights that the overlap of NMS in PD and a frequent co-occurrence (102, 103). For example, fatigue may occur alongside depression and apathy, and distinguishing between these symptoms can be challenging clinically (104, 105). Similarly, depression and apathy share symptom features such as reduced motivation and anhedonia which can make categorization of symptoms difficult (106). Despite evidence of overlapping clinical features, symptom-based classifications have the limitation of fragmenting clinically and biologically related phenomena into separate categories. While this framework remains useful for an initial clinical approach, it may limit recognition of shared mechanisms and obscure broader patterns of symptom clustering and disease expression.

### 3.2 System-based classification

A system-based models organizes NMS according to underlying pathophysiological features such as neurotransmitter systems or anatomical pathways. In the present review, we have showcased the relevance of dopaminergic, serotonergic, cholinergic, and noradrenergic systems (4, 8, 107). A system-based classification aligns with contemporary models of PD as a multisystem neurodegenerative disorder rather than one confined to dopaminergic pathways. Nevertheless, there is cumulative evidence that NMS are associated with dysfunction across shared neural circuits, including limbic and frontostriatal circuitry implicated in affective, motivational, cognitive, and autonomic symptoms (4). In fact, these networks appear to be modulated by multiple interacting neurotransmitter systems rather than a single dominant pathway (4, 8). For example, dopaminergic pathways, particularly nigrostriatal, meso-cortical and mesolimbic circuits, contribute not only to motor function but also to mood regulation, anxiety, pain, sleep disorder, cognitive impairment and bladder dysfunction (8). Similarly, serotonergic network abnormalities have also been implicated in depression, anxiety, sleep disturbances, and visual hallucinations (8). Cholinergic pathways have been implicated in motor and NMS in PD, including cognitive impairment, depression, apathy, and sleep disturbances. It has been suggested that dopamine and cholinergic pathways are linked (108). Imaging studies have documented the role of dopaminergic and cholinergic projections in cognition and behavioral functions (109).

Therefore, system-based classification may oversimplify the underlying neurobiology by attempting to assign symptoms to individual neurotransmitter systems that are, in reality, highly interconnected. As suggested by Pena-Zelayeta (4), this supports the hypothesis that symptoms of PD may be better understood through overlapping neural systems than an isolated pathophysiological mechanism.

### 3.3 Subtype- and trajectory-based models

Recent proposed models to better understand the systemic nature of PD include the identification of subtypes based on patterns of neurodegeneration (110–112).

Horsager and Borghammer (110) propose a disease model of PD called the α-Synuclein Origin site and Connectome (SOC) model (110). This model highlights two subtypes of PD based on the anatomical location of the α-synuclein pathology. In the body-first subtype, pathology is postulated to start in the peripheral nervous system, such as the enteric nervous system and spread to the sympathetic trunk and the brainstem through the autonomic nervous system. Disease progression follows a bottom-up ascending progression pattern and involves the brain at a later stage. In the “brain-first” subtype, the pathology onset is unilateral, usually involving the olfactory bulb or amygdala and spreads to ipsilateral structures such as the substantia nigra. The body-first PD subtype experiences early autonomic dysfunction and RBD, whereas the brain-first subtype may experience early neurodegeneration in the brain and later-onset autonomic dysfunction.

Furthermore, data-driven approaches involving cluster analysis have identified distinct clinical subtypes of PD (113, 114). These studies aim to group patients based on certain sets of identified symptoms, recognizing that NMS clusters often co-occur in somewhat predictable patterns (114). Mu et al. (113) identified four clusters that correspond to previous studies and include “mild,” motor-dominant,” and “severe.” (113) Lawton et al. (115) identified four new clusters in two large independent cohort of newly diagnosed patients that were associated with levodopa response and motor progression rates (115). Fereshtehnejad et al. (116) identified longitudinal clinical trajectories and brain atrophy patterns of three subtypes previously reported as “mild-motor predominant,” “intermediate,” and “diffuse-malignant.” According to this classification system, early development of mild cognitive impairment (MCI), orthostatic hypotension, and RBD, at the drug-naïve stage defines the “diffuse malignant” subtype. These patients experience the most rapid disease progression in non-motor domains and faster decline in activities of daily living during their long-term (8 year) follow up (116).

These subtype and trajectory models contribute to improving the understanding of disease pathophysiology, heterogeneity, and prognosis.

## 4 Lumping NMS domains to re-appraise current classification limitations

### 4.1 Depression, anxiety, apathy, and fatigue

Depression, anxiety, apathy, and fatigue are typically classified as distinct neuropsychiatric or behavioral domains in PD (4, 117). However, in clinical practice, these symptoms frequently co-occur and are often difficult to distinguish from one another (17, 118).

From a classification perspective, this cluster highlights a key limitation of domain-based frameworks. Although categorized separately, these symptoms demonstrate substantial phenomenological overlap, including fatigue, affect, and low motivation (17). This overlap complicates diagnostic boundaries and suggests that these symptoms may not represent fully independent nosological entities (17).

From a neurobiological perspective, this cluster of symptoms has been linked to overlapping dysfunction across dopaminergic, serotonergic, and noradrenergic systems, as well as limbic and frontostriatal circuits (17, 117, 119). While certain features may be more strongly associated with specific pathways, the overall pattern reflects distributed network dysfunction rather than discrete system involvement (17). These further challenges a system-based classification for NMS.

Treatment response provides additional insight. Selective serotonin reuptake inhibitors (SSRIs) and serotonin-norepinephrine reuptake inhibitors (SNRIs) are effective for treatment of both depressive and anxiety symptoms (17, 119, 120) while dopaminergic therapies may have variable effects on apathy (17) and fatigue (121).

Taken together, the symptom cluster of depression, anxiety, apathy, and fatigue illustrates that affective and motivational symptoms in PD are better understood in an integrated fashion rather than discrete domains, favoring a classification framework that considers shared neurobiology and clinical interdependence.

### 4.2 Cognition and psychosis

Cognitive impairment and psychosis are typically classified as separate NMS domains in PD (6, 12). However, these features frequently co-occur, particularly in later stages of the disease, and are both strongly associated with disease progression (84, 122). Their co-occurrence with advancing disease suggests that these symptoms may represent related manifestations of broader cortical and network dysfunction in the context of progressive neurodegeneration (79, 123).

From a mechanistic perspective, both cognitive impairment and psychosis are associated with cholinergic deficits, cortical Lewy body pathology, and disruption of large-scale neural networks (124–126). This challenges the symptom-based or system-based classifications as neither framework adequately captures the shared neurobiology underlying these features.

Treatment considerations further highlight the connections between cognition and psychosis. Cholinesterase inhibitors, used for cognitive impairment, may also reduce hallucinations in some patients (124). These overlapping treatment effects suggest partial convergence in underlying mechanisms, while also illustrating the difficulty of isolating symptom-specific pathways.

Taken together, cognitive impairment and psychosis in PD are better conceptualized as overlapping features of progressive cortical and network dysfunction rather than distinct clinical entities.

### 4.3 Sleep and autonomic dysfunction

Both domains involve dysfunction within brainstem and peripheral autonomic networks, as well as broader neurodegenerative processes affecting multiple systems (127, 128). RBD is strongly associated with the presence of a synucleinopathy and has been proposed as a marker of early disease (129, 130).

Treatment approaches further illustrate overlap across domains. A recent review by Iranzo et al. (131) suggests that the pharmacological management of sleep in PD is heterogeneous and pathology specific (131). It may require different therapeutic approaches depending on the underlying sleep disorder and contributing motor, autonomic, psychiatric, and medication related factors (132, 133).

Furthermore, as discussed above, Fereshtehnehad et al. (116) noted that the presence of sleep disturbance and autonomic dysfunction at disease onset is associated with rapid disease progression, early development of dementia, and shorter survival (116). Sleep and autonomic dysfunction highlight the importance of temporal progression and non-motor system involvement in PD. Their early and overlapping presentation challenges domain-based classification and are more consistent with models that incorporate disease trajectory and system-level interactions (110, 134).

### 4.4 Medication effects and impulse control disorders

Impulse control disorders (ICDs), including gambling disorder, hypersexuality, and compulsive shopping, are classified as complications of PD (135, 136). However, their relationship to both disease mechanisms and treatment effects challenges their placement within existing classification systems (137).

In a large cohort of PD patients, the prevalence of ICDs was identified to be 13.6% (138). ICDs are strongly associated with the use of dopamine agonist therapy (139, 140). While they are often grouped within neuropsychiatric domains, their development is more prevalent in patients treated with dopamine agonists, suggesting it less likely from the intrinsic disease progression alone (138).

From a mechanistic perspective, ICDs are associated with dopaminergic overstimulation of mesolimbic reward pathways (138). However, individual factors, such as younger age, is a risk factor for developing ICDs, suggesting that underlying disease-related vulnerability and treatment effects interact to produce these behaviors.

This interplay highlights a limitation, which does not distinguish between disease-related and treatment-induced phenomena. While some NMS may arise directly from neurodegeneration, others are influenced by treatment modalities used for PD, such as dopaminergic agents (15).

Treatment of one symptom domain may lead to changes in another, either improving or exacerbating symptoms. For example, dopamine agonists, may cause hallucinations and impulse control disorders such as pathological gambling (141). These observations highlight the complexity of disentangling disease-related and treatment-related effects (17).

Treatment of ICDs often involves lowering or discontinuing dopaminergic therapy, which may improve behavioral symptoms but exacerbate motor symptoms (135) or other NMS such as apathy (142). This further underscores the interconnected nature of symptom domains and the difficulty of isolating discrete categories.

ICDs illustrate that a comprehensive classification of NMS cannot rely only on symptom description without considering background clinical factors like medication effects. A more integrated approach that accounts for both intrinsic and iatrogenic factors may provide a more accurate representation of NMS in PD.

## 5 Closing remarks and future steps

### 5.1 Relevance of emerging models

The evolving landscape of PD subtyping has increasingly incorporated data – driven, biomarker-informed, and trajectory-based frameworks (111, 143). For example, machine learning-driven models have gained attention for their ability to identify clinically meaningful PD patient subtypes through the integration of clinical data (111, 144). Newer emerging subtyping approaches have combined clinical, imaging, genetic, and other biomarker data to capture disease heterogeneity that may be missed by single-domain classification (143, 145).

The integration of multi-omics platforms, including genomic, transcriptomic, metabolomic, and proteomic data, has become increasingly important in disease stratification and personalized medicine (146). These approaches have shown promise for more refined patient risk stratification (147) and personalized therapeutic strategies (146).

Dynamic subtyping and staging approaches aim to account for temporal variability in disease expression and progression (148, 149). Longitudinal modeling approaches may allow subtype categories to be refined as the disease evolves and new data become available, improving the ability to track disease trajectories over time (150, 151). Dynamic and staging-oriented approaches have been investigated in psychiatry, where traditional diagnostic categories may not adequately capture illness progression over time (148, 152).

The use of real-world data in phenotyping and subtyping frameworks is also gaining attention, particularly because the population used for clinical trials may not accurately represent the heterogeneity seen in clinical practice (153). Such models may improve external validity and implementation feasibility (153). However, the variability in the quality of data collected and standardization may limit is widespread use (153).

### 5.2 Implications for clinical practice

The limitations of current classification systems have practical implications for clinical care. Recognizing that NMS frequently co-occur and, in some cases, form identifiable clusters may improve clinical reasoning and patient management (114, 154, 155). For instance, the identification of distinct symptom clusters may inform more targeted treatment selection, as interventions aimed at improving one domain may impact other symptoms. An integrated approach that considers disease progression and the impact of therapeutic interventions may better reflect the clinical complexity of PD (156).

Compared with strictly domain-based classification systems, the conceptualization of disease heterogeneity may provide a more tailored framework for clinical decision-making (110, 157). Recognizing the multidimensional nature of PD, including its motor, non-motor, and treatment-related features, may support more individualized approaches to patient care (157). This perspective may help clinicians navigate management strategies as interventions targeting one domain may influence others.

An integrated approach that considers symptom overlap, temporal progression, and treatment context may assist clinicians with providing more personalized care as compared to management based on domain-based classification.

### 5.3 Positioning within existing literature

Recent reviews have emphasized the prevalence, burden, and clinical management of NMS in PD (157). Some have proposed network-based approaches (4). However, these works have largely focused on describing NMS or redefining their importance within PD.

In contrast, the present review focuses specifically on evaluating the frameworks developed to classify NMS. By examining how NMS are grouped and interpreted, this work highlights gaps limitations that are less frequently addressed in the literature and emphasizes the need for models that better reflect clinical and biological complexity.

### 5.4 Limitations

The present review is narrative and is therefore subject to selection bias as there was no formal process to assess the quality of the included articles. However, given the limited number of studies challenging the current classification of NMS in PD, a narrative review is valuable as it proposes alternative classification strategies. Additionally, the clusters of NMS presented in this review were formed based on available knowledge of underlying pathophysiology, treatment response, and clinical experience, and do not reflect a data-driven approach to prospectively validate these concepts.

### 5.5 Future directions

Future research should focus on improving early diagnosis of NMS in PD through biomarker development and identifying disease-modifying strategies, as current approaches remain largely symptomatic (4, 157). Some have suggested that personalized treatment strategies based on phenotypes may help manage NMS in PD (4). Future work should also refine subtype-based models and clarify the relationship between genetic, environmental, and prodromal markers and disease trajectories (110).

## 6 Conclusions

In this narrative review, we conclude that multiple NMS can overlap in both clinical presentations and underlying neurobiology, concur along the disease course, and are variably influenced by treatment effects. An integrated classification system is required to incorporate these features, currently not addressed in the commonly used, domain-based classification systems.
